# Inhibitory Effect of Fermented *Flammulina velutipes* Polysaccharides on Mice Intestinal Inflammation

**DOI:** 10.3389/fnut.2022.934073

**Published:** 2022-06-21

**Authors:** Sheng Ma, Jianxiong Xu, Ting Lai, Weina Xu, Jing Zhang, Hongcai Zhang, Weiyi Zhang

**Affiliations:** ^1^School of Agriculture and Biology, Shanghai Jiao Tong University, Shanghai, China; ^2^Shanghai Key Laboratory for Veterinary and Biotechnology, Shanghai, China; ^3^Shanghai Center of Agri-Products Quality and Safety, Shanghai, China

**Keywords:** *Flammulina velutipes* polysaccharides, solid anaerobic fermentation, anti-inflammatory capacities, NLRP3 signaling pathway, antioxidant capacities

## Abstract

To investigate the effect of *Flammulina velutipes* polysaccharides (FVPs) on mice intestinal inflammation, FVPs were extracted from *Flammulina velutipes* (FV) using a solid anaerobic fermentation technique. The antioxidant and anti-inflammatory capacities of FVP and fermented FVP (FFVP) induced by lipopolysaccharide (LPS) were investigated *in vitro* and *in vivo*. The results showed that the yield of FFVP (9.44%) was higher than that of FVP (8.65%), but the molecular weight (MW) of FFVP (15,702 Da) was lower than that of FVP (15,961 Da). The antioxidant and anti-inflammatory capacities of FFVP were higher than that of FVP in preventing mice diarrhea, enhancing antioxidant capacities, and reducing the secretion and mRNA expression of interleukin-1β (IL-1β), IL-6, IL-18, and tumor necrosis factor-α (TNF-α). The anti-inflammatory mechanisms of FVP and FFVP were analyzed by inhibiting the activation of the NLRP3 signaling pathway using an LPS-induced mice model. This study indicated that FFVP could be used as a functional antioxidant, indicating a potential application in functional food and health products.

## Highlights

- First report the extraction of fermented *Flammulina velutipes* polysaccharides (FFVP).
- FFVP with low molecular weight has higher anti-inflammatory capacities than FVP.
- FFVP regulates inflammatory response by inhibiting NLRP3 signal pathway activation.

## Introduction

*Flammulina velutipes* (FV), as one of the four major cultivated edible fungi, is widely artificially cultivated in the world (1). It is well-known that FV mainly contains abundant polysaccharides and proteins, and among them, FV polysaccharides (FVP) have shown many excellent biological activities, including antioxidant, antimicrobial, anti-aging, anti-tumor, and immunomodulatory properties (2, 3). For instance, previous studies have reported that FVP could improve antioxidant capacities and anti-inflammatory properties by increasing the immunoglobulin and immune factors in mice serum (4, 5), and play an immunomodulatory role in regulating the intestinal environment and improving the balance of flora (6).

It is well-known that the preparation and purification of polysaccharides are critical to investigating their functional capacities. The traditional extraction methods of polysaccharides include hot water extraction (HWE), enzyme extraction (EE), chemistry assisted extraction (CAE), and pressurized liquid extraction (PLE) (7). However, the low extraction efficacy, high cost, and environmental pollution are the pitfalls of these methods. Recently, microbial fermentation has been widely employed to extract bioactive compounds and is applied in food, agriculture, environment, and medicine fields (8). Moreover, the solid-state fermentation, one of the most common forms of microbial fermentation, is an alternative method to produce large amounts of fungal polysaccharides, which has the advantages of a shorter production cycle, less environmental pollution, higher yield, and better product quality (9, 10). For instance, polysaccharides were extracted from *Monascus purpureus* using microbial fermentation, which enhanced immunomodulatory capacities by significantly improving pinocytic and phagocytic capacities (11). Moreover, polysaccharides were extracted from *Inonotus hispidus* using solid-state fermentation, showing that they had high yields and antioxidant capacities and could reduce H_2_O_2_-induced oxidative damage to cells *in vitro* (12).

NLRP3 (Nucleotide- binding oligomerization domain, leucine- rich repeat and pyrin domain- containing 3) inflammasome is a multiprotein complex located in the cytoplasm and has been proven to be closely related to the pathological process of intestinal inflammation (13). Cui et al. (14) reported that colitis could be ameliorated by polysaccharides from *Scutellaria baicalensis Georgi via* suppressing NLRP3 inflammasome activation. Thus, FVP has excellent antioxidant and anti-inflammatory capacities. There has few research on the extraction of fermented FVPs and their antioxidant and anti-inflammatory capacities. Therefore, we hypothesized that FVP extracted using microbial fermentation has higher extraction efficacy and biological functions than unfermented ones *in vitro* and *in vivo*. In this work, the yields and molecular weight (MW) of FFVP and unfermented FVP were compared, and anti-inflammatory capacities were studied using lipopolysaccharide (LPS)-induced mice model. This study could provide a new idea for developing a functional additive to alleviate intestinal inflammation.

## Materials and Methods

### Materials and Reagents

*Flammulina velutipes* was obtained by Shanghai Guangming Senyuan Biotechnology Co., Ltd., China. The microbial culture starter, including *Bacillus subtilis* CMCCB 63501, *Bifidobacterium longum* ATCC 15707, and *Saccharomyces cerevisiae* ATCC 9763, was a kind gift from Shanghai Chuangbo Ecological Engineering Co., Ltd., China (production batch No. CB08190529). LPS was obtained from Sigma–Aldrich (St. Louis, MO, United States). Other chemicals and reagents were of analytical grade and were purchased from Sigma–Aldrich (St. Louis, MO, United States).

### Extraction of FFVP

*Flammulina velutipes* was pulverized using Waring blender (Shanghai Shibang Machinery Co., Ltd., China) and passed through a 0.75 mm sieve. Microbial culture starter (0.10%, v/w) was then added to the smashed FV and stirred (200 rpm) in a fermentation tank (Shanghai xiaohan industrial development co., Ltd., Shanghai, China) at the ambient room temperature. The optimal fermentation conditions, including the amounts of molasses contents (3%), temperature (28°C), moisture contents (40%), and culture time (10 days), were previously reported for the production of FFVP (15).

Ultrahigh pressure (HPP600/5L, Suzhou, China)–ultrasonic extraction method (HA131–50–01, Nantong supercritical extraction Co., Jiangsu, China) was used to extract the FVP and FFVP from FV and FFV, respectively. The optimized extraction conditions, including pH = 5, ultrahigh pressure of 360 MPa, ultrahigh pressure time of 5 min, ultrasonic power of 180 W, extraction temperature of 80°C, and extraction time of 40 min, have been reported based on a previous study (15). During the FVP extraction, lipids, pigments, and small molecular compounds were all washed out from FV first. The residues were then extracted with distilled water, and the supernatant was filtered, combined, and concentrated. The crude polysaccharides were then precipitated by adding ethanol and washed successively with anhydrous ethanol, acetone, and petroleum ether (16). The extracted crude FVP was then harvested after deproteinization with Sevag solution (chloroform: butylalcohol = 5:1) and fractionated through diethylaminoethyl (DEAE)–Sepharose fast flow column (elution conditions: distilled water and 0.05, 0.1, 0.2, 0.3, and 0.5 mol/L NaCl gradient solutions) (1).

### Molecular Weight Determination

The MW of FVP and FFVP was determined using HPLC (Waters Arc HPLC, Shanghai, China) equipped with a diode array and differential detector. Analyses were performed on a charged surface hybrid (CSH) C^18^ column (5 μm, 4.6 × 150 mm). The treatment conditions consisted of a mobile phase: 0.1% formic acid aqueous solution (A) and methanol (B), a flow rate of 0.6 ml/min, and an injection volume of 20 μl, respectively. A standard curve with the elution volume plotted against the logarithm of MW was constructed using the Dextran T standards and glucose according to Tang's method (17), and the MW of FVP/FFVP was calculated by the calibration curve equation based on their elution volume (18).

### Animals and Experimental Design

Four-week-old male BALB/c mice obtained from Shanghai JieSiJie Laboratory Animals Co., Ltd. (Shanghai, China) were put in polypropylene cages. All mice were raised under standard conditions of 12 h light/dark cycle at 25 ± 2°C, with a relative humidity of 55 ± 5%. The mice had free access to a basal diet (Jiangsu Xietong Inc., Nanjing, China) and water. After one week adaption, 60 mice were randomly divided into 6 groups with 10 mice in each group and filled the stomachs with 2 mL sample everyday as follows: CON group (normal saline), LPS group (normal saline), LFVP group (50 mg/kg FVP), HFVP group (100 mg/kg FVP), LFFVP group (50 mg/kg FFVP) and HFFVP group (100 mg/kg FFVP). The experiment lasted for 60 days. On the last day, the CON group was injected intraperitoneally with normal saline (2 ml), while the LPS group, LFVP group, HFVP group, LFFVP group, and HFFVP group were injected intraperitoneally with LPS (2 ml, 3 mg/kg) to induce intestinal inflammation in mice (19). All experimental procedures were approved by the Institutional Animal Ethics Committee of Shanghai Jiao Tong University under animal protocol number 202101207.

### Behavior and Appearance Observation of Mice

A total of three mice were randomly selected from each group and placed in a box after intraperitoneal injection. The behavior and appearance, including diarrhea occurrence and mice hair, were observed after 4 h (20).

### Serum Oxidative Stress and Inflammatory Factor Analysis

Blood was collected from the eyeball of mice under anesthesia, and then the serum was obtained after centrifugation (5420, Eppendorf, Hamburg, Germany; 4,000 rpm, 10 min). The H_2_O_2_ and malondialdehyde (MDA) levels, catalase (CAT), glutathione peroxidase (GSH-Px), superoxide dismutase (SOD) capacities, and total antioxidant capacities (T-AOC) in serum were detected using an assay kit (Beyotime Biotechnology, Shanghai, China). Besides, the levels of interleukin-1β (IL-1β), IL-6, IL-18, and tumor necrosis factor-α (TNF-α) in serum were detected by enzyme linked immunosorbent assay method (ELISA) using ELISA Kit (Nanjing Jiancheng Bioengineering Institute, Nanjing, China).

### Histological Analysis of Mice Intestine

The jejunum, ileum, and colon tissue sections were fixed in 10% formalin and embedded in paraffin to analyze the pathological changes. Sections (5 μm thick) were stained with a hematoxylin and eosin (H&E) stain kit (D006-1-1, Nanjing Jiancheng Bioengineering Institute, Nanjing, China) and observed under a light microscope (Eclipse Ci-L, Nikon, Tokyo, Japan). Image-Pro Plus 6.0 (Media Cybernetics, Rockville, MD, United States) was used for analysis. The height of intact villi and the depth of crypts in each section were measured, respectively, and the ratio of villi height to crypt depth (V/C value) was calculated (*n* = 5).

### Quantitative Real-Time Polymerase Chain Reaction Analysis

Total RNA of intestinal samples was isolated with Trizol Reagent (Takara, Kyoto, Japan) after incubation for 24 h (21). RNA amount and quality were determined using the spectrophotometer (GeneQuant 1300 GE, Austin, TX, United States). The absorbance of samples between 1.8 and 2.0 at 260-280 nm was subjected to acceptable quality and integrity. The cDNA was synthesized with TransScript First-Strand complementary DNA synthesis SuperMix (AT341-01, Roche, Basel, Switzerland). The primer sequences are shown in Supplementary Table 1. Polymerase chain reaction (PCR) was performed in StepOnePlus Real-time PCR system (ABI, Carlsbad, CA, United States) using SYBR Green PCR Core Reagents (Roche, Basel, Switzerland). The housekeeping gene, β-actin, was used as a reference gene for normalization. Data were analyzed according to the 2^−ΔΔCt^ method. Results were expressed as relative mRNA levels.

### Western Blot Analysis

The intestinal samples were lysed in radio immunoprecipitation assay (RIPA) buffer (Beyotime, Shanghai, China) with protease and phosphatase inhibitor cocktails (Sigma Co., St. Louis, MO, United States) for 10 min on a rocker at 4°C. Proteins of samples were analyzed using sodium dodecyl sulfate polyacrylamide gel electrophoresis (SDS-PAGE) and then transferred onto polyvinylidene fluoride (PVDF) membranes (0.45 μm, Millipore, Billerica, MA, United States). Each PVDF membrane was blocked with tris buffered saline tween (TBST; 100 mM Tris-HCl,150 mM NaCl, 0.05% Tween 20, pH 7.5) with 5% non-fat dried milk for 2 h, and then incubated with the following primary antibodies: NLRP3 (1:1,500), ASC (1:100), Caspase-1 (1:800), IL-1β (1:400), and β-actin (1: 5,000, Sungene biotech, China) overnight at 4°C on a shaker (SC390C, Shanghai Brave Construction Development co., Ltd., Shanghai, China). The goat anti-rabbit of 1:3000 (ZSGE-BIO, China) horse radish peroxidase (HRP) conjugate secondary antibody was then added and the WB results was observed through electrochemical luminescence (ECL) imaging system after adding the enhanced ECL reagent (Beyotime, Shanghai, China). The β-actin was used as a protein loading control. Quantitative analysis was carried out using Amersham Imager 600 (Cytiva, Montana, United States).

### Statistical Analysis

All analysis was performed with the SPSS 22.0 software (SPSS Incorporated, Armonk, NY, United States), and statistical significance was analyzed using one-way ANOVA followed by an LSD test as a post-test. Data were expressed as mean ± standard error, and all measurements were conducted in triplicate. The values of *P* < 0.05 and *P* < 0.01 were considered significant and markedly significant, respectively, in different treatment groups.

## Results and Discussions

### Physicochemical Analysis of FVP and FFVP

**Figure 1:**
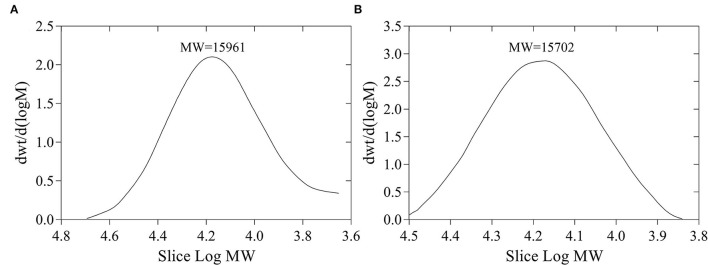
Molecular weight (MW) of FVP **(A)** and FFVP **(B)**. FVP and FFVP are *Flammulina velutipes* polysaccharides and fermented *Flammulina velutipes* polysaccharides.

The yields of FVP and FFVP were 8.65 and 9.44%, respectively, and MW of 15,961 and 15,702 Da (Figure 1), respectively. Besides, the MW/MN (Weight-average Molecular Weight/Number-average Molecular Weight) of FVP and FFVP were 1.20 and 1.09 (Supplementary Figure 2), respectively, which was similar to a previous report (1.042) (16). A previous study has also reported that the contents and MW of tea polysaccharides, compared with the unfermented group, were, respectively, increased and decreased after fermentation treatment, and the biological capacities of fermented fractions were also better than unfermented ones (22). For instance, the MW of *Nostoc commune Vauch* polysaccharides (NCVP) was higher than that of fermented ones, while the antioxidant capacities were significantly improved during *in vitro* digestion (23). Thus, microbial fermentation treatment could increase extraction yield and decrease MW of FVP, and the reason could be that one or more glycosidic bonds of FVP may be broken during the fermentation (24, 25).

### Behavior and Appearance Observation of Mice

Diarrhea is a life-threatening condition and could cause extreme loss of fluid and salts from the animal body (26). Compared with the CON group, the mice of the LPS group had diarrhea with shaggy hair (Supplementary Figure 1). All of the LFVP group, HFVP group, LFFVP group, and HFFVP group did not have diarrhea, and only the LFVP group was shaggy. Besides, the mice of the CON group and HFFVP group were vigorous with shiny hair, while others gathered in the corner. A previous study has also reported that diarrhea could be prevented using plant polysaccharides including banana, guar, and soya polysaccharides (27). For instance, polysaccharides from *S. chinensis* could prevent diarrhea in rats by improving the ultrastructure of the gut (28). Besides, the polysaccharides from *Alpiniae oxyphyllae* and *Pogostemon cablin* also decreased diarrhea by inhibiting porcine epidemic diarrhea virus (PEDV) reproduction (29, 30).

### Serum Antioxidant Capacities and Inflammatory Factor Contents of FVP and FFVP

**Figure 2:**
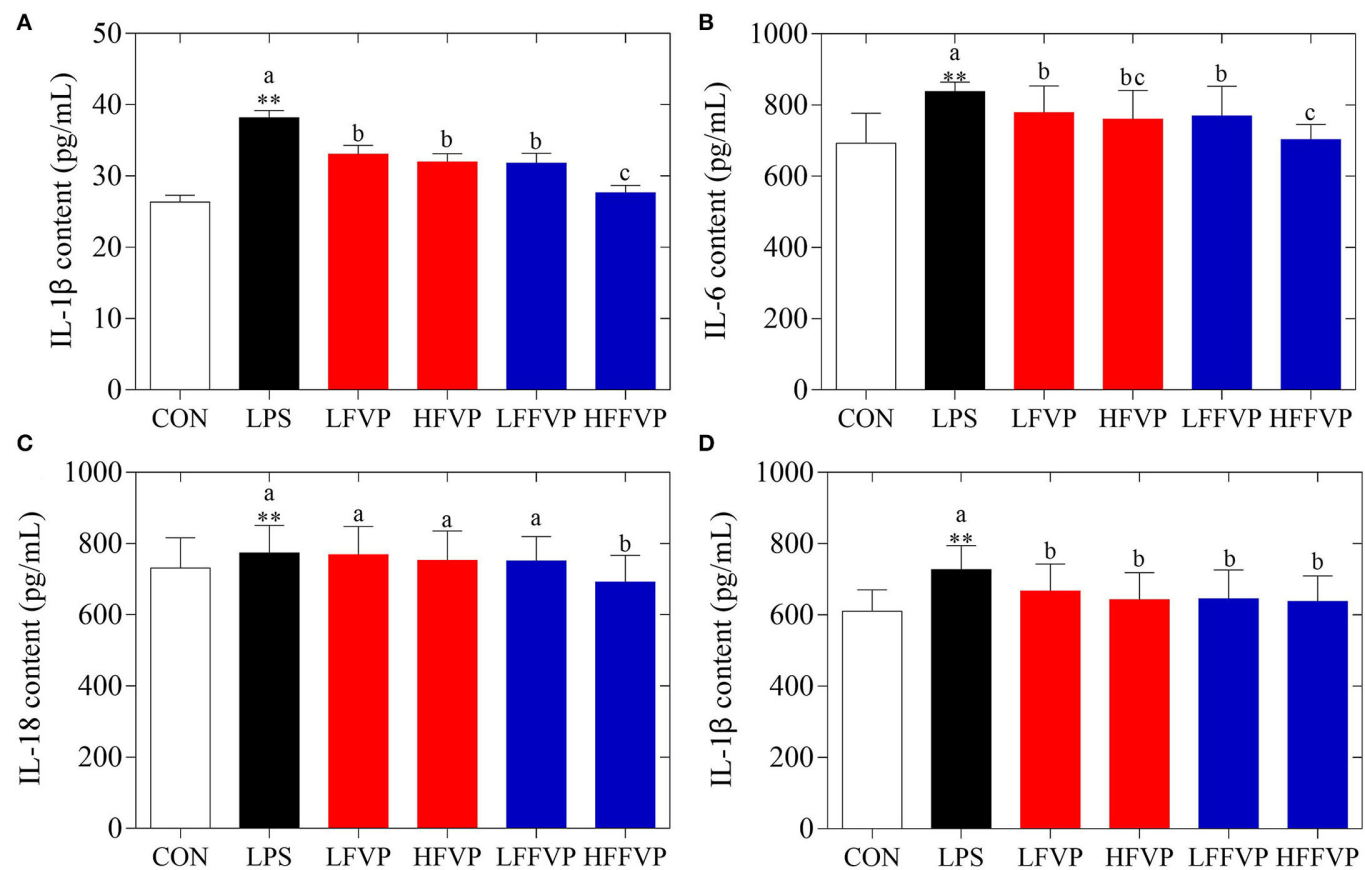
Serum inflammatory factor contents, including IL-1β **(A)**, IL-6 **(B)**, IL-18 **(C)**, and TNF-α **(D)**. Mice were stimulated with 3 mg/kg LPS and/or 50 and 100 mg/kg FVP and FFVP. CON group, LPS group (3 mg/kg LPS), FVP and FFVP groups (50/100 mg/kg FVP and FFVP plus 3 mg/kg LPS). FV, *Flammulina velutipes*; FVP, *Flammulina velutipes* polysaccharides; CON, control group; LPS, lipopolysaccharide; LFVP, low dose FVP group; HFVP, high dose FVP group; LFFVP, low dose FFVP group; HFFVP, high dose FFVP group. **P < 0.01 represents LPS vs. CON group; a, b and c (*P* < 0.05) represent FVP and FFVP groups vs. LPS treated mice.

Serum antioxidant capacities of FVP and FFVP are shown in Supplementary Table 2. Compared with the CON group, H_2_O_2_ (3.66 nmol/ml) and MDA (7.64 nmol/ml) contents of LPS group's serum was increased significantly (*P* < 0.05), while the CAT (0.49 U/ml), GSH-Px (1,319.61 U/ml), SOD (226.87 U/ml), and T-AOC (1.23 U/ml) were decreased significantly (*P* < 0.05). The antioxidant capacities gradually increased after FVP and FFVP gavage. Serum inflammatory factor contents of FVP and FFVP are shown in Figure 2. Compared with the CON group, the contents of IL-1β, IL-6, IL-18, and TNF-α in the LPS group's serum were increased significantly (*P* < 0.05). For instance, the IL-1β contents of the LPS group (38.17 pg/ml) were 1.45 times more than the CON group (26.34 pg/ml). Serum inflammatory factor content of the LFVP group, HFVP group, LFFVP group, and HFFVP group decreased significantly (*P* < 0.05) compared with the LPS group, whose IL-1β contents were 33.05, 31.97, 31.83, and 27.65 pg/ml, respectively. Besides, IL-1β, IL-6, and IL-18 contents of the HFFVP group were lower than that of the HFVP group.

Wang et al. (31) reported the antioxidant and anti-inflammatory effects of *Gynostemma pentaphyllum herb* polysaccharides (GPP) in diabetic mice, and the results indicated that GPP could enhance SOD, CAT and GSH-Px capacities, as well as decreasing MDA and IL-6 contents, which was consistent to the present study. Moreover, the antioxidant and anti-inflammatory activities of FFVP were also better than that of FVP. Jiang et al. (32) also reported that low MW *Enteromorpha prolifera* polysaccharides could enhance anti-inflammatory capacities and decrease inflammatory response through multiple signaling pathways, including TLR2/NF-κB, PKC/ERK/MAPK, and PI3K/Akt pathways (33, 34). Besides, low MW polysaccharides had higher antioxidant capacities than high MW ones (35).

### Histological Analysis of Mice Intestine

**Figure 3:**
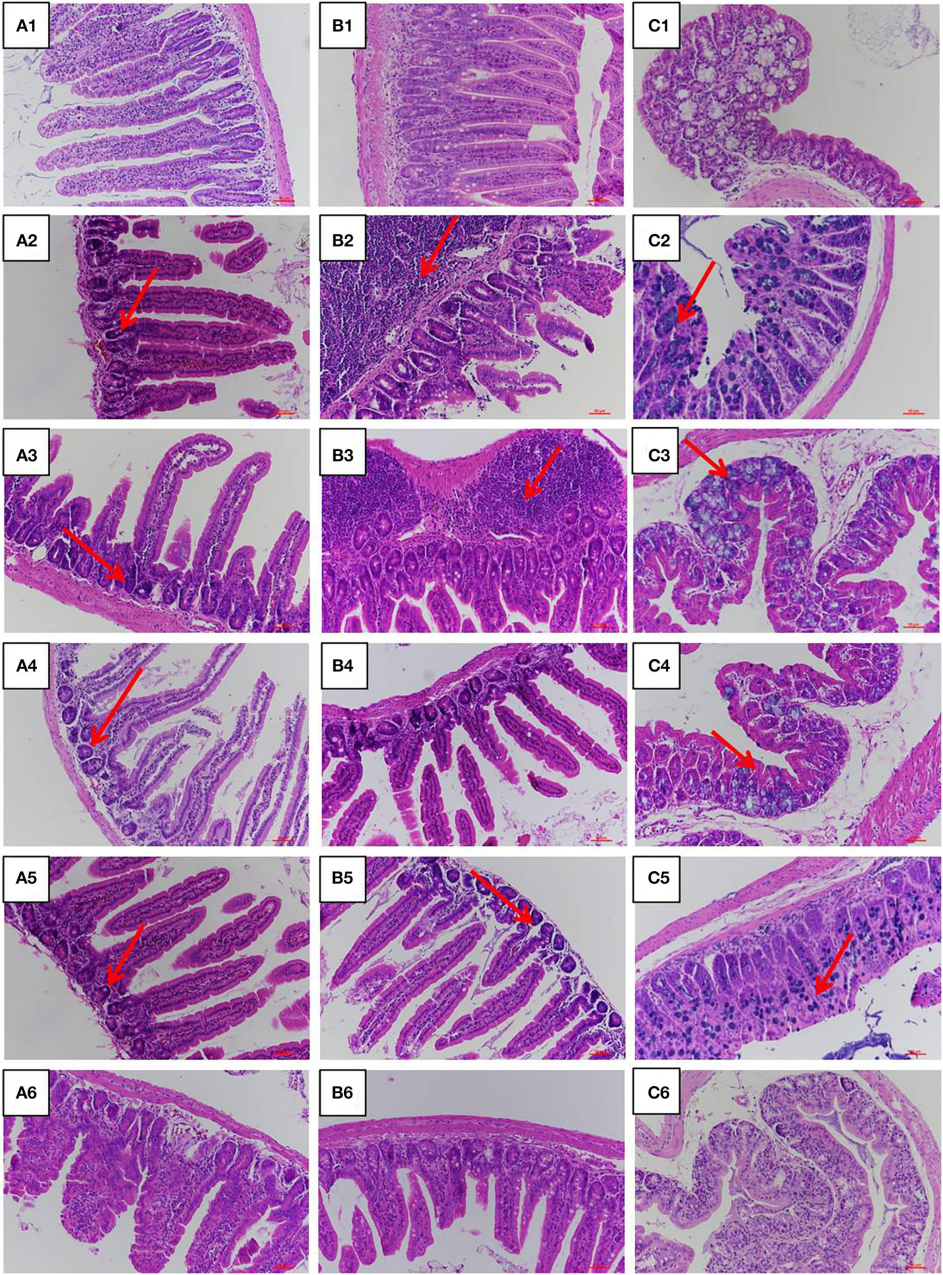
Jejunum **(A)**, ileum **(B)**, and colon **(C)** intestinal morphology of CON group (1), LPS group (2), LFVP group (3), HFVP group (4), LFFVP group (5), HFFVP group (6). The magnification is 20 ×. FV, *Flammulina velutipes*; FVP, *Flammulina velutipes* polysaccharides; CON, control group; LPS, lipopolysaccharide; LFVP, low dose FVP group; HFVP, high dose FVP group; LFFVP, low dose FFVP group; HFFVP, high dose FFVP group.

The jejunum, ileum, and colon intestinal morphology are shown in Figure 3 and the ratio of villus height to crypt depth (V/C value) is show in Table 1. For the LPS group, intestinal epithelial tissue structure was dissolved, most of the inflammatory cells were infiltrated, and mucosa was severely damaged with a large range of ulcers. Compared with the LPS group, intestinal epithelial tissue and intestinal villi were improved in the LFVP group, HFVP group, LFFVP group, and HFFVP group to varying degrees. Meanwhile, intestinal inflammatory damage in the LFVP and HFVP groups were more serious than that in the LFFVP and HFFVP groups, respectively. A previous study showed that FVP could ameliorate bowel inflammation and modulate the gut microbiota on dextran sulfate sodium (DSS)-induced inflammatory bowel disease (36). Besides, FVP could also improve the clinical symptoms and attenuate the mRNA and protein expressions of inflammatory cytokines and oxidative markers in colon tissue.

**Table 1:** The ratio of villus height to crypt depth (V/C value).

	**Group**	**Villus height (μm)**	**Crypt depth (μm)**	**V/C value**
Jejunum	CON	354.50 ± 35.24^a^	62.30 ± 2.86^e^	5.69 ± 1.23^a^
	LPS	206.56 ± 21.37^e^	99.23 ± 6.02^a^	2.08 ± 0.31^d^
	LFVP	241.62 ± 23.94^d^	93.26 ± 5.68^b^	2.59 ± 0.17^d^
	HFVP	281.47 ± 23.58^c^	88.29 ± 4.06^b^	3.19 ± 0.32^c^
	LFFVP	260.05 ± 24.31^d^	90.44 ± 4.27^b^	2.88 ± 0.26^d^
	HFFVP	283.19 ± 31.45^c^	85.42 ± 4.31^c^	3.32 ± 0.51^c^
Ileum	CON	238.96 ± 31.25^a^	60.38 ± 6.15^c^	3.96 ± 0.58^a^
	LPS	123.23 ± 24.98^e^	89.72 ± 8.24^a^	1.37 ± 0.09^d^
	LFVP	142.67 ± 25.01^d^	86.22 ± 7.51^a^	1.65 ± 0.74^cd^
	HFVP	161.57 ± 24.14^c^	78.71 ± 7.18^b^	2.05 ± 0.14^b^
	LFFVP	149.90 ± 25.67^d^	84.33 ± 7.04^a^	1.78 ± 0.82^c^
	HFFVP	165.88 ± 27.36^c^	78.04 ± 7.23^b^	2.13 ± 0.36^b^
Colon	CON	341.61 ± 36.18^a^	61.02 ± 2.86^e^	5.60 ± 1.20^a^
	LPS	229.58 ± 26.49^e^	95.61 ± 6.02^a^	2.40 ± 0.12^d^
	LFVP	257.12 ± 19.84^d^	91.03 ± 5.68^b^	2.82 ± 0.25^d^
	HFVP	275.13 ± 12.04^c^	86.04 ± 4.16^b^	3.20 ± 0.87^c^
	LFFVP	268.71 ± 22.57^d^	85.64 ± 4.27^b^	3.14 ± 1.03^d^
	HFFVP	284.37 ± 21.24^c^	82.77 ± 4.19^c^	3.44 ± 0.69^c^

*The same letters (a–e) in the same column indicate no significant difference (P > 0.05), while the different letters in the same column indicate significant difference (P < 0.05). CON, control group; LFVP, low dose FVP group; HFVP, high dose FVP group; LFFVP, low dose FFVP group; HFFVP, high dose FFVP group*.

### mRNA Expression of Inflammatory Factors

**Figure 4:**
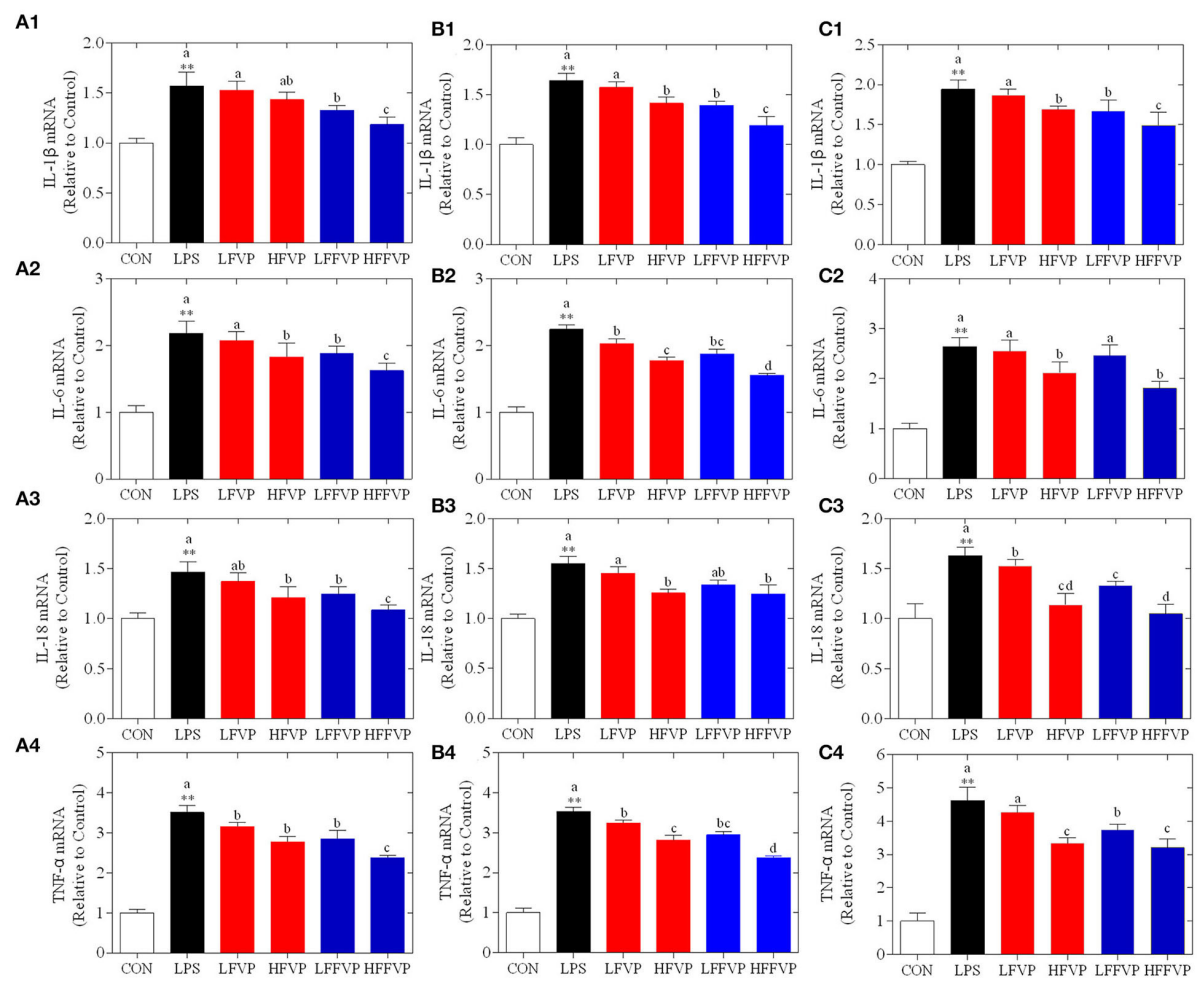
LPS-induced IL-1β (1), IL-6 (2), IL-18 (3) and TNF-α (4) mRNA expression treated by FVP and FFVP in jejunum **(A)**, ileum **(B)**, and colon **(C)**. Mice were stimulated with 3 mg/kg LPS and/or 50 and 100 mg/kg FVP and FFVP. CON group, LPS group (3 mg/kg LPS), FVP and FFVP groups (50/100 mg/kg FVP and FFVP plus 3 mg/kg LPS). FV, *Flammulina velutipes*; FVP, *Flammulina velutipes* polysaccharides; CON, control group; LPS, lipopolysaccharide; LFVP, low dose FVP group; HFVP, high dose FVP group; LFFVP, low dose FFVP group; HFFVP, high dose FFVP group. **P < 0.01 represents LPS vs. CON group; a, b and c (*P* < 0.05) represent FVP and FFVP groups vs. LPS treated mice.

Lipopolysaccharide-induced IL-1β, IL-6, IL-18, and TNF-α mRNA expression treated by FVP and FFVP in jejunum, ileum, and colon are shown in Figure 4. The mRNA expression of inflammatory factors in the LPS group was significantly increased compared with the CON group (*P* < 0.05). The LFVP group, HFVP group, LFFVP group, and HFFVP group's mRNA expression of inflammatory factors were lower than that of the LPS group. For instance, IL-1β mRNA expression of the LPS group in the colon was 1.94, while in the LFVP group, HFVP group, LFFVP group, and HFFVP group were 1.87, 1.69, 1.67, and 1.49, respectively. Besides, HFVP's mRNA expressions of all four inflammatory factors were significantly higher than that of HFFVP's (*P* < 0.05). Han et al. (37) also reported that *Gracilaria Lemaneiformis* polysaccharides could prevent colitis in Balb/c mice and inhibited pro-inflammatory cytokines expression to attenuate acute inflammation, which was consistent with this study. Therefore, FVP and FFVP play an anti-inflammatory effect by inhibiting pro-inflammatory cytokines mRNA expression.

### Protein Expression of NLRP3 Signaling Pathway

**Figure 5:**
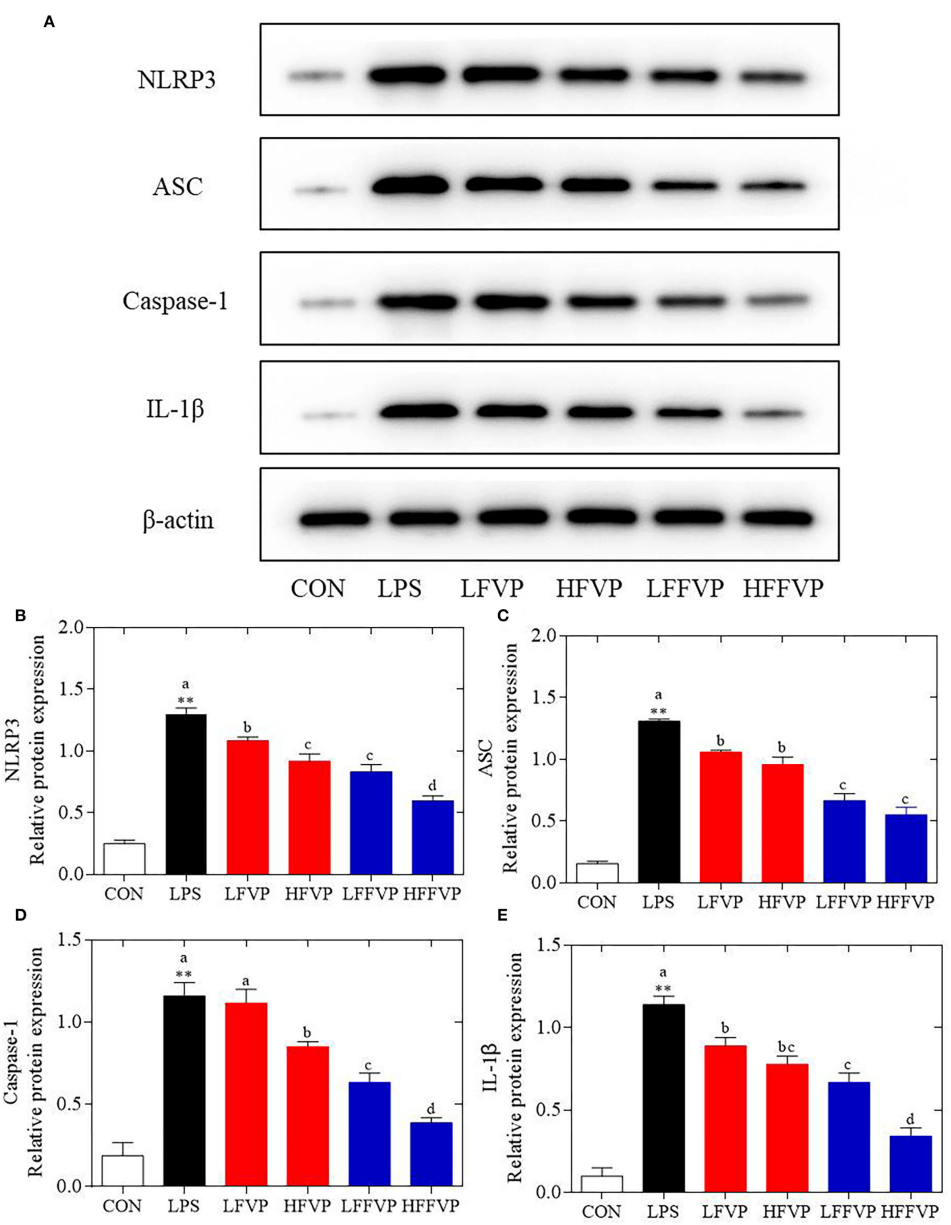
LPS-induced NLRP3/β-actin **(A,B)**, ASC/β-actin **(A,C)**, Caspase-1/β-actin **(A,D)** and IL-1β/β-actin **(A,E)** protein expression treated by FVP and FFVP. FV, *Flammulina velutipes*; FVP, *Flammulina velutipes* polysaccharides; CON, control group; LPS, lipopolysaccharide; LFVP, low dose FVP group; HFVP, high dose FVP group; LFFVP, low dose FFVP group; HFFVP, high dose FFVP group. **P < 0.01 represents LPS vs. CON group; a, b and c (*P* < 0.05) represent FVP and FFVP groups vs. LPS treated mice.

The NLRP3 signaling pathway was one of the most important anti-inflammatory signaling pathways (38). It could be activated by LPS and mediated by immune response of host to microbial infection and cell injury via agglomeration of NLRP3 inflammasome, resulting in producing activated Caspase-1 and mature IL-1β and IL-18 (39). As the colon was sensitive to LPS-induced inflammatory response, the colon was selected for the subsequent Western Blot test. Compared with the CON group, protein expression of NLRP3, ASC, Caspase-1, and IL-1β were significantly increased in the LPS group. Protein expressions of LFVP, HFVP, LFFVP, and HFFVP groups were lower than that of the LPS group. At the same dose, protein expression of FFVP group was significantly lower than that of FVP group (Figure 5).

Pan et al. (40) reported that polysaccharides from *Smilax china* L. could ameliorate ulcerative colitis by inhibiting the galectin-3/NLRP3 inflammasome pathway. Besides, *Chayote* polysaccharides could also reduce active Caspase-1 protein levels and downregulate NLRP3 and IL-1β gene expression (41), suggesting that the NLRP3 inflammasome activation was dependent on the processing of inactive pro-Caspase-1 to active Caspase-1, which enhances IL-1β release, reactive oxygen species (ROS) production, and pyroptosis, an inflammatory form of programmed cell death (42). In this work, protein expression of NLRP3 ASC, Caspase-1 and IL-1β were significantly increased in the LPS group compared with CON group. Besides, these protein expression decreased after treated by FVP/FFVP compared with LPS group. Thus, the anti-inflammatory capacities of FVP and FFVP were also inhibited *via* activation of an NLRP3 signaling pathway.

## Conclusion

The FFVP has higher antioxidant and anti-inflammatory capacities than FVP in preventing diarrhea, enhancing antioxidant capacities, and reducing the secretion and mRNA expression of IL-1β, IL-6, IL-18, and TNF-α. Besides, the anti-inflammatory capacities of FVP and FFVP are to inhibit the activation of an NLRP3 signaling pathway. This study indicated that FFVP could be used as functional additives and confered to its beneficial functions such as antioxidant and anti- inflammation.

## Data Availability Statement

The original contributions presented in the study are included in the article/Supplementary Material, further inquiries can be directed to the corresponding author/s.

## Ethics Statement

The animal study was reviewed and approved by Institutional Animal Ethics Committee of Shanghai Jiao Tong University.

## Author Contributions

SM, WX, JZ, WZ, and JX: design and conceptualization. SM, HZ, and JX: manuscript writing and proofreading. SM, TL, WX, JZ, HZ, WZ, and JX: experimental work, data analysis, and interpretation. JX: funding acquisition. All authors have read and agreed to the published version of the manuscript.

## Funding

This research was funded by Shanghai Agriculture Applied Technology Development Program, China (Grant No. X2019-02-08-00-08-F01155) and National Natural Science Foundation of China (Grant No. 31872367).

## Conflict of Interest

The authors declare that the research was conducted in the absence of any commercial or financial relationships that could be construed as a potential conflict of interest.

## Publisher's Note

All claims expressed in this article are solely those of the authors and do not necessarily represent those of their affiliated organizations, or those of the publisher, the editors and the reviewers. Any product that may be evaluated in this article, or claim that may be made by its manufacturer, is not guaranteed or endorsed by the publisher.

## Abbreviations

FV: *Flammulina velutipes*
FVP: *Flammulina velutipes* polysaccharides
FFVP: fermented *Flammulina velutipes* polysaccharides
LFVP: low dose FVP group
HFVP: high dose FVP group
LFFVP: low dose FFVP group
HFFVP: high dose FFVP group
LPS: lipopolysaccharide
SDS-PAGE: sodium dodecyl sulfate polyacrylamide gel electrophoresis
PVDF: polyvinylidene fluoride
TBST: Tris buffered saline tween
RIPA: radio immunoprecipitation assay
ECL: electrochemical luminescence
HWE: hot water extraction
EE: enzyme extraction
CAE: chemistry assisted extraction
PLE: pressurized liquid extraction
MW: molecular weight
MDA: Malondialdehyde
CAT: catalase
GSH-Px: Glutathioneperoxidase
SOD: Super oxide dismutase
T-AOC: total antioxidant capacity
H&E: hematoxylin and eosin
V/C: villi height to crypt depth
HRP: horse radish peroxidase
NCVP: *Nostoc commune Vauch* polysaccharides
PEDV: porcine epidemic diarrhea virus
GPP: *Gynostemma pentaphyllum herb* polysaccharides.
