# Supplementary material for: Inhibitory Effect of Fermented Flammulina velutipes Polysaccharides on Mice Intestinal Inflammation
Source: Front Nutr. 2022 Jun 21;9:934073. doi: 10.3389/fnut.2022.934073 (PMC9277576; doi:10.3389/fnut.2022.934073)
Supplement: Supplementary file 1 [file Data_Sheet_1.pdf]

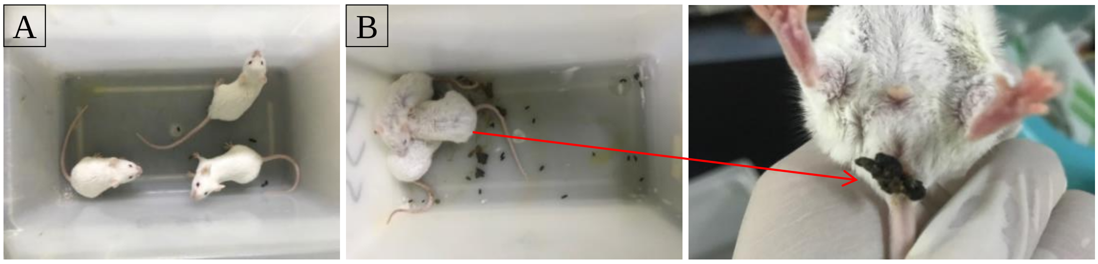

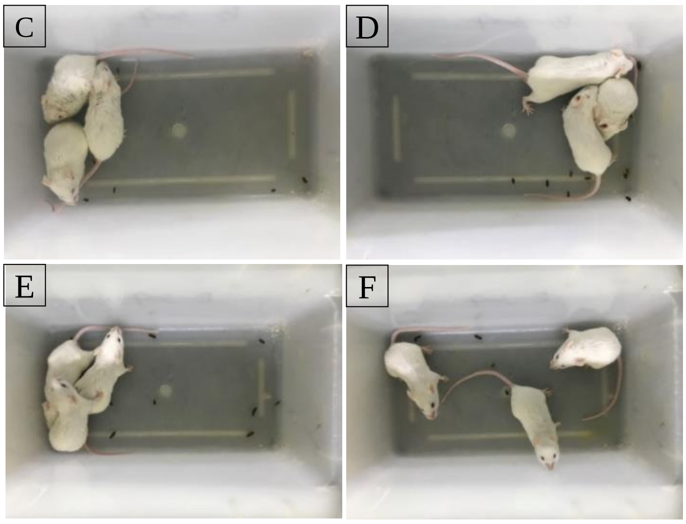

Group	Diarrhea	Shaggy
A	×	×
B	✓	✓
C	×	✓
D	×	×
E	×	×
F	×	×

Supplementary Fig.1. Behavior and appearance observation of mice from CON group (A), LPS group (B), LFVP group (C), HFVP group (D), LFFVP group (E), HFFVP group (F).

Mice were stimulated with 3 mg/kg LPS and/or 50 and 100 mg/kg FVP/FFVP. CON group, LPS group (3 mg/kg LPS), FVP and FFVP groups (50/100 mg/kg FVP/FFVP plus 3 mg/kg LPS).

FV: *Flammulina velutipes*; FVP: *Flammulina velutipes* polysaccharides; CON: control group; LPS: lipopolysaccharides; LFVP: low dose FVP group; HFVP: high dose FVP group; LFFVP: low dose FFVP group; HFFVP: high dose FFVP group.

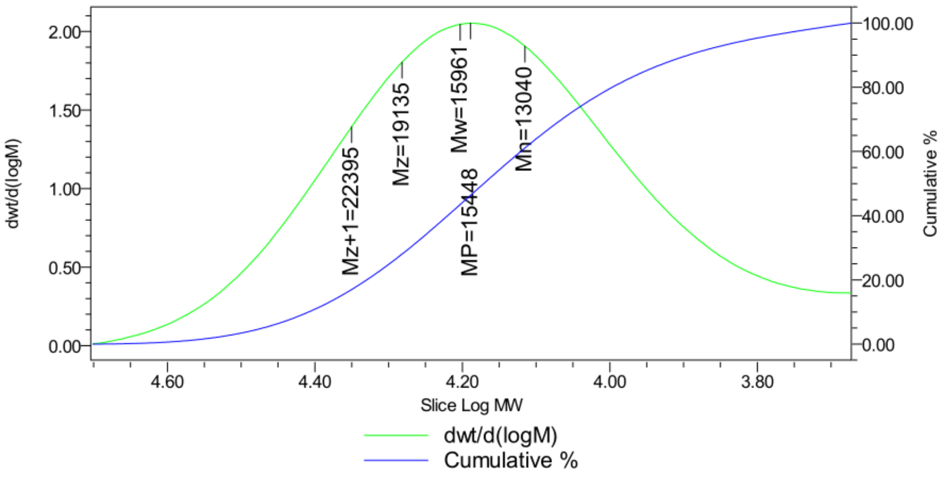

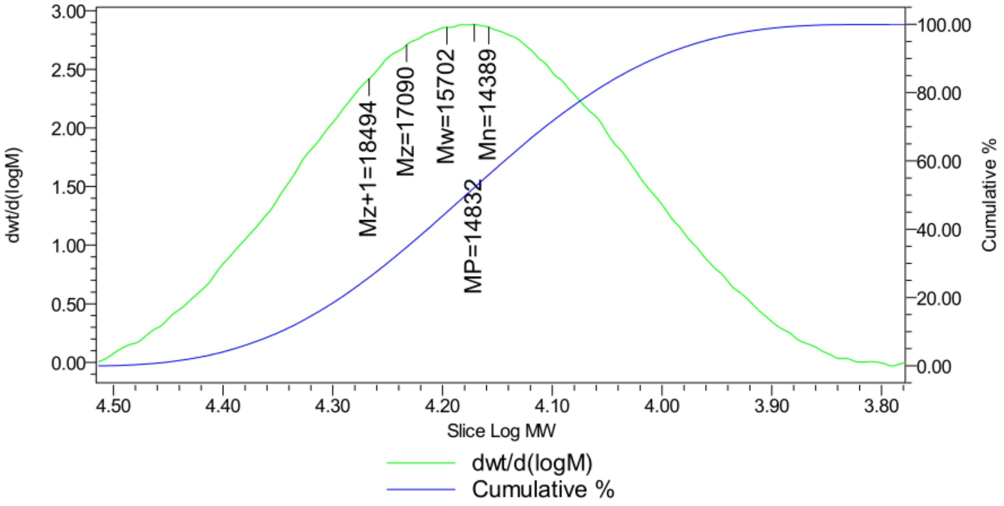

Supplementary Fig.2. Molecular weight (MW) of FVP and FFVP from original report.

FVP and FFVP are *Flammulina velutipes* polysaccharides and fermented *Flammulina velutipes* polysaccharides.

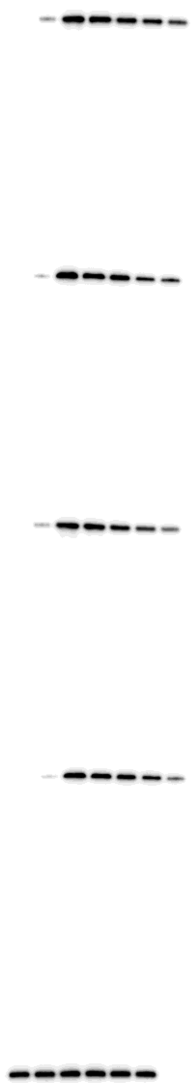

Supplementary Fig.3. LPS-induced NLRP3, ASC, Caspase-1, IL-1β and β-actin protein expression treated by FVP and FFVP.

Supplementary Table 1 Primer sequences

Genes	Gene Number	Primer Sequences
IL-1β	NM_008361.4	For: TTGAAGTTGACGGACCCCAA<br>Rev: TACTGCCTGCCTGAAGCTC
IL-6	NM_001314054.1	For: GATGGATGCTACCAAATGGA<br>Rev: CCAGGTAGCTATGGTACTCCAGAA
IL-18	NM_001357221.1	For: CATCAGGACAAAGAAAGCCG<br>Rev: AGTTGTCTGATTCCAGGTCTCCAT
TNF-α	NM_001278601.1	For: CCTCTAGCCCACGTCGTAGC<br>Rev: AGCAATGACTCCAAAGTAGACC
β-actin	NM_007393.5	For: TGCTATGTTGCTCTAGACTTCG<br>Rev: ATGCCACAGGATTCCATACC

IL-1β: Interleukin-1β; IL-6: Interleukin-6; IL-18: Interleukin-18; TNF-α: Tumor necrosis factor-α.

Supplementary Table 2 Serum antioxidant capacities

Group	H_2O_2 (mmol/L)	MDA (nmol/mL)	CAT (U/mL)	GSH-Px (U/mL)	SOD (U/mL)	T-AOC (U/mL)
CON	2.04 ± 0.13^{e}	3.85 ± 0.27^{e}	1.76 ± 0.11^{a}	1487.46 ± 112.71^{a}	261.35 ± 23.91^{a}	4.04 ± 0.15^{a}
LPS	3.66 ± 0.32^{a}	7.64 ± 0.59^{a}	0.49 ± 0.07^{e}	1319.61 ± 98.65^{c}	226.87 ± 21.09^{d}	1.23 ± 0.09^{c}
LFVP	3.17 ± 0.28^{b}	6.39 ± 0.52^{b}	0.95 ± 0.18^{d}	1433.68 ± 124.31^{b}	243.18 ± 24.58^{c}	3.63 ± 0.26^{b}
HFVP	3.01 ± 0.35^{b}	6.11 ± 0.51^{b}	1.05 ± 0.14^{d}	1445.61 ± 120.89^{b}	251.03 ± 22.07^{b}	3.97 ± 0.38^{a}
LFFVP	2.93 ± 0.22^{b}	5.22 ± 0.46^{b}	1.17 ± 0.21^{d}	1490.28 ± 128.14^{a}	248.36 ± 24.86^{c}	4.18 ± 0.42^{a}
HFFVP	2.88 ± 0.24^{c}	5.05 ± 0.33^{c}	1.27 ± 0.28^{c}	1497.45 ± 110.57^{a}	254.61 ± 20.04^{b}	4.45 ± 0.23^{a}

In the same column, values with same superscript letter (a-e) were not significantly different ($P > 0.05$); values with different superscript letter were significantly different ($P < 0.05$).

FV: *Flammulina velutipes*; FVP: *Flammulina velutipes* polysaccharides; CON: control group; LPS: lipopolysaccharides; LFVP: low dose FVP group; HFVP: high dose FVP group; LFFVP: low dose FFVP group; HFFVP: high dose FFVP group.
